# Establishment of a *Wolbachia* Superinfection in *Aedes aegypti* Mosquitoes as a Potential Approach for Future Resistance Management

**DOI:** 10.1371/journal.ppat.1005434

**Published:** 2016-02-18

**Authors:** D. Albert Joubert, Thomas Walker, Lauren B. Carrington, Jyotika Taneja De Bruyne, Duong Hue T. Kien, Nhat Le Thanh Hoang, Nguyen Van Vinh Chau, Iñaki Iturbe-Ormaetxe, Cameron P. Simmons, Scott L. O’Neill

**Affiliations:** 1 School of Biological Sciences, Monash University, Clayton, Melbourne, Victoria, Australia; 2 Oxford University Clinical Research Unit, Wellcome Trust Major Overseas Programme, Hospital for Tropical Diseases, Ho Chi Minh City, Vietnam; 3 Department of Microbiology and Immunology, University of Melbourne at the Peter Doherty Institute, Parkville, Melbourne, Victoria, Australia; 4 Hospital for Tropical Diseases, Ho Chi Minh City, Vietnam; Stanford University, UNITED STATES

## Abstract

*Wolbachia pipientis* is an endosymbiotic bacterium estimated to chronically infect between 40–75% of all arthropod species. *Aedes aegypti*, the principle mosquito vector of dengue virus (DENV), is not a natural host of *Wolbachia*. The transinfection of *Wolbachia* strains such as *w*AlbB, *w*Mel and *w*MelPop-CLA into *Ae*. *aegypti* has been shown to significantly reduce the vector competence of this mosquito for a range of human pathogens in the laboratory. This has led to *w*Mel-transinfected *Ae*. *aegypti* currently being released in five countries to evaluate its effectiveness to control dengue disease in human populations. Here we describe the generation of a superinfected *Ae*. *aegypti* mosquito line simultaneously infected with two avirulent *Wolbachia* strains, *w*Mel and *w*AlbB. The line carries a high overall *Wolbachia* density and tissue localisation of the individual strains is very similar to each respective single infected parental line. The superinfected line induces unidirectional cytoplasmic incompatibility (CI) when crossed to each single infected parental line, suggesting that the superinfection would have the capacity to replace either of the single constituent infections already present in a mosquito population. No significant differences in fitness parameters were observed between the superinfected line and the parental lines under the experimental conditions tested. Finally, the superinfected line blocks DENV replication more efficiently than the single *w*Mel strain when challenged with blood meals from viremic dengue patients. These results suggest that the deployment of superinfections could be used to replace single infections and may represent an effective strategy to help manage potential resistance by DENV to field deployments of single infected strains.

## Author Summary

Dengue fever is a viral disease transmitted by *Aedes aegypti* mosquitoes and more than 30% of the world’s population is at risk. The control of dengue virus (DENV) transmission has been problematic as no vaccines or drugs are effective against the four serotypes. Vector control of mosquitoes during epidemics is considered the only option to prevent transmission. Recently, a novel biocontrol method using the endosymbiotic bacterium *Wolbachia* has been developed in which DENV replication is significantly inhibited in *Wolbachia*-infected *Ae*. *aegypti*. This bacterium also induces a reproductive phenotype called cytoplasmic incompatibility that allows rapid invasion of uninfected mosquito populations. Like any control method, evolutionary responses are expected of the system that might limit its future effectiveness. Here we report the generation and characterization of a superinfected *Ae*. *aegypti* line containing two *Wolbachia* strains (*w*Mel and *w*AlbB). We show that stable *Wolbachia* superinfections are more effective at blocking dengue than single infections. Superinfections also demonstrate a cytoplasmic incompatibility phenotype that should enable them to replace single infections in the field. This represents a potential mechanism for resistance management in regions where single infections have already been deployed.

## Introduction

The endosymbiotic bacterium *Wolbachia pipientis* was first discovered in 1924 by Marshall Hertig and Burt Wolbach in ovaries of the mosquito *Culex pipiens* [1]. *Wolbachia* is a Gram-negative, obligate endosymbiont that is maternally transmitted [2]. It is estimated that around 40–75% of all arthropod species are infected with *Wolbachia* [3, 4] and the phenomenal success of this bacterium has been attributed to its ability to manipulate the reproductive biology of its host to provide it with a vertical transmission advantage in host populations [5]. These manipulations include feminization, parthenogenesis, cytoplasmic incompatibility (CI) and male-killing [6, 7]. Of these reproductive phenotypes, CI is probably the best studied and describes the phenomenon of early embryonic death resulting from crosses between an infected male and uninfected female or in crosses involving two different *Wolbachia* strains [7, 8].

More recently, *Wolbachia* has been shown to limit pathogen replication, in particular the enveloped, positive single-stranded RNA viruses such as dengue (DENV), yellow fever (YFV) and chikungunya (CHIKV) [9–12]. *Wolbachia* also inhibits additional human pathogens transmitted by mosquitoes including filarial nematodes [13] and malaria parasites [14–16]. The mechanism of pathogen inhibition by *Wolbachia* is still being investigated, but blocking has been linked to priming of the host innate immune system and competition for limited resources between pathogens and *Wolbachia* [17, 18].

The ability of *Wolbachia* to limit pathogen replication has led to the field deployment of *Ae*. *aegypti* transinfected with two Drosophila *Wolbachia* strains, *w*Mel and *w*MelPop-CLA [19, 20]. *w*MelPop-CLA is a pathogenic strain that grows to high densities in insect hosts and infected adult insects have significantly reduced lifespan [21]. In contrast, the closely related *w*Mel strain is avirulent and grows to a lower density in most insect tissues. Correspondingly, total DENV inhibition in whole adult *w*Mel-infected mosquitoes is lower than in *w*MelPop-CLA infected mosquitoes [12]. However, key to the success of such an approach is the use of *Wolbachia* strains that can successfully invade wild mosquito populations through the action of CI. The *w*MelPop-CLA *Wolbachia* strain imposes significant fitness costs to *Aedes* mosquitoes including reducing fecundity and egg longevity [9, 12, 22, 23]. Although the *w*MelPop-CLA strain has a stronger inhibitory effect on total DENV replication in whole mosquito bodies, the significant fitness costs were predicted to prevent invasion of wild mosquito populations [24]. Semi-field cage experiments revealed that the *w*Mel strain would likely invade wild mosquito populations at a faster rate than the virulent *w*MelPop-CLA strain [12]. Based on these findings, the *w*Mel strain was released into two suburbs of Cairns, Australia in 2011 and reached fixation in mosquito populations within a few months [19].

The avirulent *Wolbachia* strain *w*AlbB, transinfected from closely related *Aedes albopictus* mosquitoes, also inhibits DENV replication in *Ae*. *aegypti* with smaller fitness costs than *w*MelPop-CLA [25]. If avirulent *Wolbachia* strains such as *w*Mel or *w*AlbB induce the most favourable phenotypic effects for establishment in wild mosquito populations, the potential long-term development of resistance to the inhibitory effects on DENV must be considered. A strategy to overcome the potential development of DENV resistance to either the *w*Mel or *w*AlbB strains in wild mosquito populations is to release a superinfected line that would ‘sweep over’ the existing single infection. In this study, we describe the generation of an *Ae*. *aegypti* mosquito line co-infected with *Wolbachia* strains *w*Mel and *w*AlbB. The CI attributes of this superinfected line, named *w*Mel*w*AlbB, indicate the superinfection should replace either single infection in a population and as such provide a potential mechanism to address resistance if it were to develop. In addition, the superinfected strain shows fitness costs compatible with a successful field deployment and inhibition of DENV that is predicted to have a large impact on dengue transmission in human populations.

## Results

### *Wolbachia* density in superinfected adult female *Ae*. *aegypti* mosquitoes

**Fig 1:**
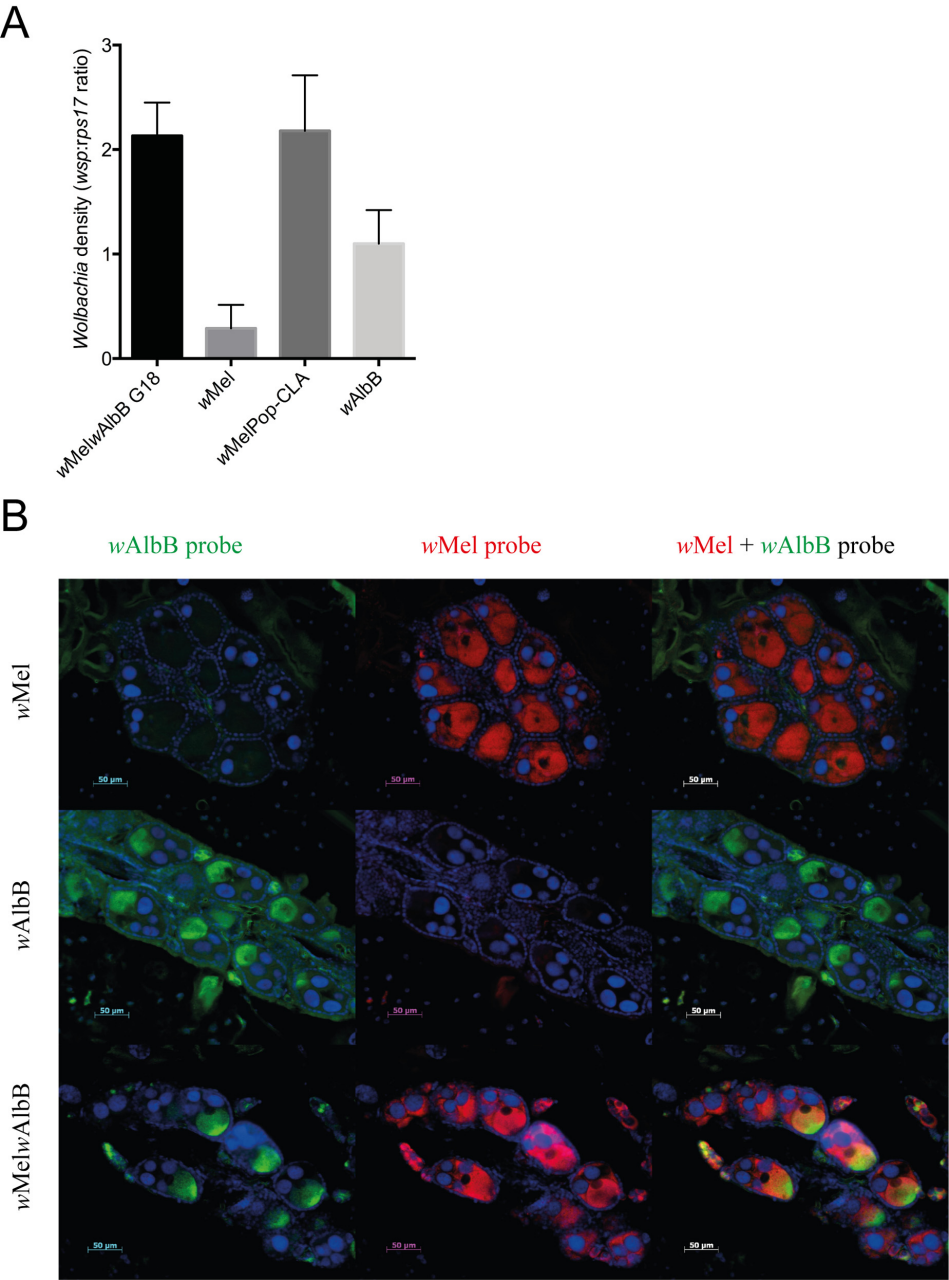
*Wolbachia* density and distribution in the *Ae*. *aegypti* superinfected line. (A) Comparison of total *Wolbachia* density between *w*Mel*w*AlbB (G18), *w*Mel, *w*AlbB and *w*MelPop-CLA infected mosquitoes. Density is expressed as the mean ratio between the *w*Mel or *w*AlbB *wsp* gene and the *Ae*. *aegypti* host *rps17* gene. Standard error of the mean is indicated (n = 10). (B) *Wolbachia* distribution in the ovaries of *w*Mel, *w*AlbB and *w*Mel*w*AlbB infected adult female mosquitoes. *Wolbachia* was visualised using FISH with probes specific to *w*Mel (red) and *w*AlbB (green). DNA is stained in blue using DAPI.

Total *Wolbachia* density in the superinfected *Ae*. *aegypti* line was determined using qPCR and primers specific for the gene encoding the *Wolbachia* surface protein (*wsp*) in conjunction with the *Ae*. *aegypti rps17* gene to ‘normalise’ for differences in mosquito size. After infection densities had stabilized by generation 18 (G18), the total *Wolbachia* density in the *w*Mel*w*AlbB line was higher than in either parental line and comparable to the virulent *w*MelPop-CLA strain (Fig 1A).

The tissue localization within adult female mosquitoes of both the *w*Mel and *w*AlbB *Wolbachia* strains in the superinfected line was determined by fluorescence *in situ* hybridisation (FISH) in formaldehyde-fixed, paraffin-embedded tissue sections using specific probes against *w*Mel (labelled in red) and *w*AlbB (labelled in green) (Fig 1B). The *Wolbachia* tissue tropism in the superinfected line was compared with the *w*Mel and *w*AlbB strains in the parental, single infected lines. We confirmed the specificity and lack of cross-reactivity of the *w*Mel and *w*AlbB FISH probes by using both probes against each of the parental lines. No *Wolbachia* signal was detected in *w*AlbB mosquitoes when using the *w*Mel probe, and *vice versa*.

Our FISH studies demonstrated the coexistence of both strains in various tissues within the adult female mosquito body. As expected for maternally transmitted symbionts, both *w*Mel and *w*AlbB strains were particularly abundant in the ovaries (Fig 1B). In addition, both strains were also found to co-localise in somatic tissues such as fat body, nervous tissue (e.g. thoracic ganglia), Malpighian tubules and salivary glands (S1 Fig). The density of *w*AlbB in all these tissues was similar in the *w*Mel*w*AlbB line as in the single *w*AlbB-infected line. However, *w*Mel was more abundant in the Malpighian tubules, fat body and muscle from the super infected line than in the parental *w*Mel line. The density of *w*Mel in salivary glands appeared to be similar in the super infected *Ae*. *aegypti* line as in the single *w*Mel line.

Interestingly, the *w*Mel and *w*AlbB *Wolbachia* strains showed quite distinct localisation patterns in ovaries of superinfected *w*Mel*w*AlbB line females. Whereas *w*Mel was found evenly distributed throughout the whole egg chamber (nurse cells and oocyte), *w*AlbB was concentrated in the posterior end of the egg chamber that contains the oocyte (Fig 1B). This is similar to the tropism observed in each the parental lines (Fig 1B). These differences in tropism could represent different patterns of binding of these two strains to the host microtubules and dynein as well as kinesin-1 that appear to drive the movement of *Wolbachia* into the oocyte during oogenesis [26, 27].

### Cytoplasmic incompatibility and maternal transmission

Maternal transmission was determined from crosses between *w*Mel*w*AlbB infected females and uninfected wild type males. We observed 100% maternal transmission for *w*AlbB across all generations and 97%, 98% and 100% transmission for *w*Mel across generations G12, G14 and G17 respectively (Table 1).

**Table 1:** Maternal transmission rates of *w*Mel and *w*AlbB in the superinfected *w*Mel*w*AlbB *Ae*. *aegypti* line.

Generation # of superinfected line	# Positive progeny—*w*AlbB	# Progeny screened—*w*AlbB	# Positive progeny—*w*Mel	# Progeny screened—*w*Mel
**G12**	88 (100%)	88	85 (97%)	88
**G14**	64 (100%)	64	63 (98%)	64
**G17**	64 (100%)	64	64 (100%)	64

Females of the superinfected line at G12, G14 and G17 were crossed to wild type males and their progeny were screened for the presence of *w*AlbB and *w*Mel by qPCR.

Cytoplasmic incompatibility (CI) was determined by setting up a series of reciprocal crosses between wild type, *w*Mel, *w*AlbB and *w*Mel*w*AlbB infected mosquitoes. Viable offspring from each of the crosses was used to determine the level of CI induced by the *w*Mel*w*AlbB line. Egg hatch rate percentages from different crosses are summarised in Table 2. Crosses between *w*Mel*w*AlbB infected females and wild type males as well as males infected with *w*Mel, *w*AlbB and *w*Mel*w*AlbB resulted in viable offspring while the reciprocal crosses resulted in no viable offspring.

**Table 2:** Cytoplasmic incompatibility (CI) between *Wolbachia* infected and wild type mosquitoes.

		Males			
		WT	*w*Mel	*w*AlbB	*w*Mel*w*AlbB
**Females**	**WT**	95.1 ± 1%	0%	0%	0.1%
	***w*Mel**	62.3 ± 4%	77.5 ± 6%	0%	3.0 ± 2%
	***w*AlbB**	77.8 ± 4%	0%	72.0 ± 4%	5.1 ± 2%
	***w*Mel*w*AlbB**	82.4 ± 2%	58.9 ± 4%	67.8 ± 5%	66.8 ± 6%

CI was determined by quantifying viable eggs resulting from a series of crosses between *Wolbachia* infected and wild type mosquitoes. Mean hatching rates are reported and standard error of the mean is indicated.

### Mosquito fitness costs of the superinfected *w*Mel*w*AlbB line

**Fig 2:**
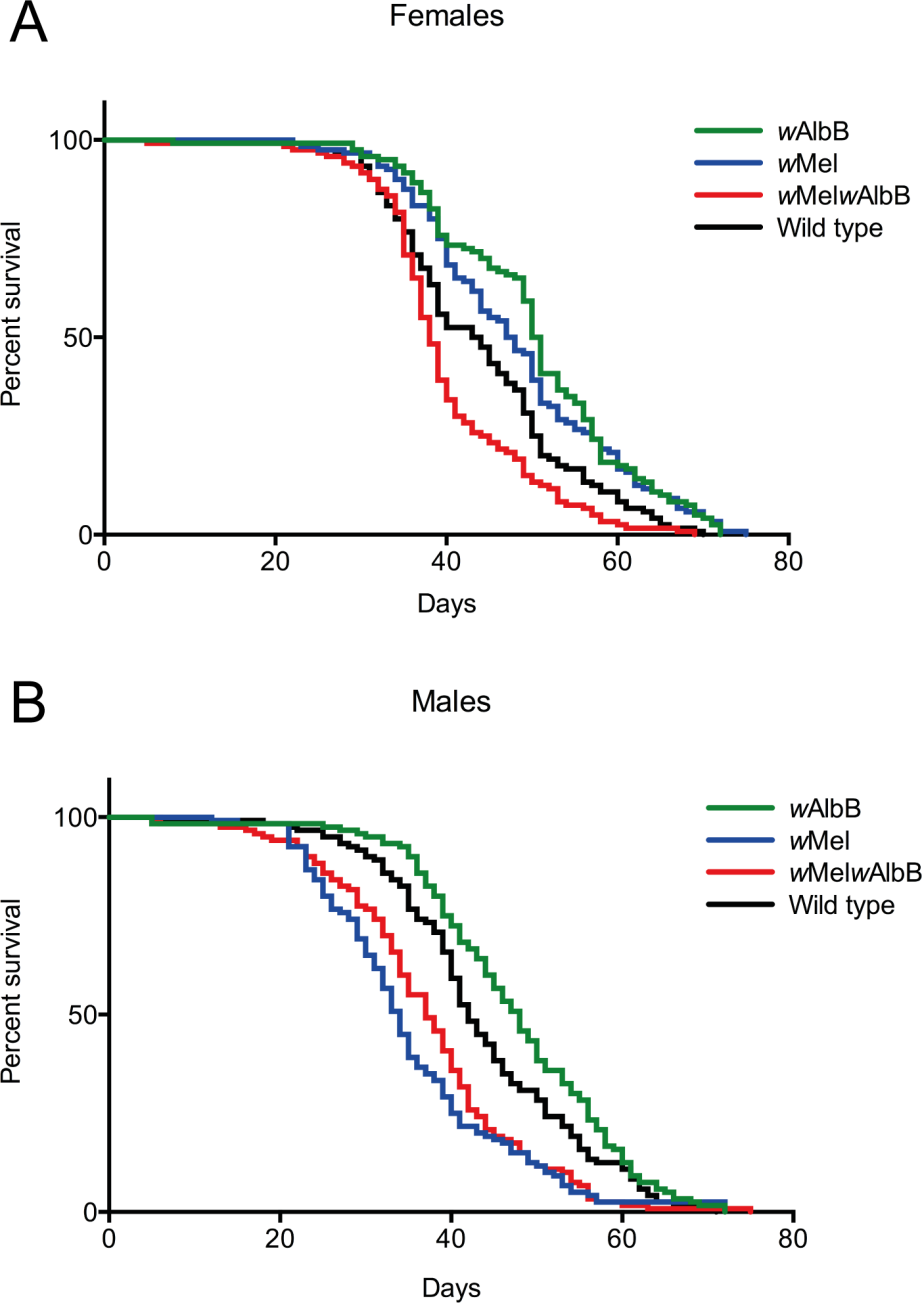
Survival plots comparing the adult lifespan of wild type *Ae*. *aegypti w*Mel, *w*AlbB and *w*Mel*w*AlbB females (A) and males (B). Wild type mosquitoes are indicated in black, *w*Mel in blue, *w*AlbB in green and *w*Mel*w*AlbB in red. All experiments were conducted using mosquitoes from G8 of the superinfected line.

**Fig 3:**
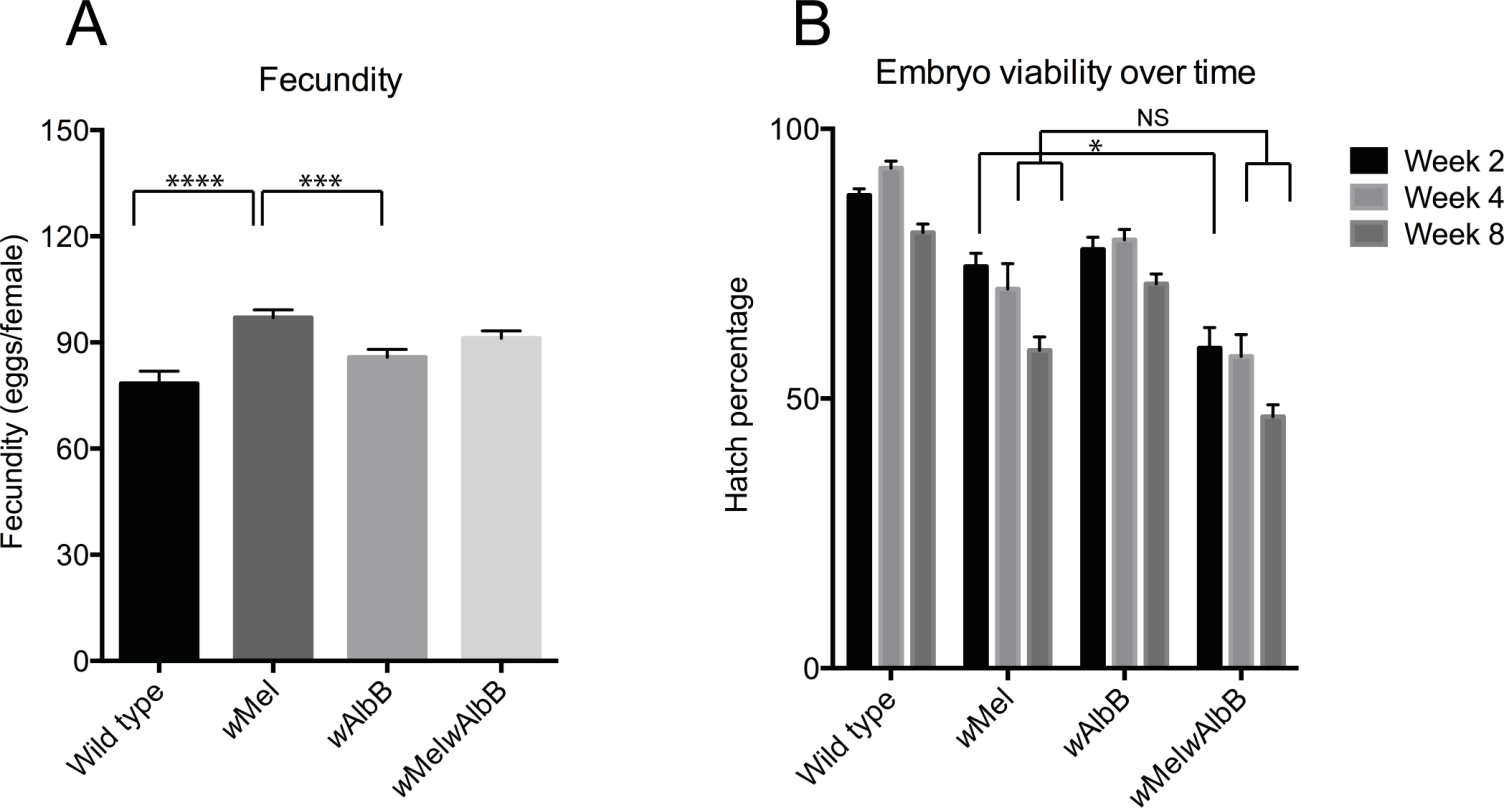
Fecundity and fertility of *Wolbachia*-infected *Ae*. *aegypti*. (A) Fecundity of *Wolbachia*-infected and wild type females as determined by mean egg production from individual female mosquitoes. Statistical significant differences between all data sets were determined using a Kruskal-Wallis test with Dunn’s multiple comparisons test. Significant differences are indicated by **** (p < 0.0001) or *** (p < 0.001) and standard error of the mean is indicated. Non-significant differences are not indicated. (B) Mean hatch rate of *Wolbachia*-infected and wild type females as determined by percentage of eggs hatching over time. Statistical differences between all data sets were determined using a two-way ANOVA with Tukey’s multiple comparisons test. For simplicity, only the comparison between *w*Mel (current release strain) and *w*Mel*w*AlbB is shown in the figure. A small but significant difference between the hatch rates for *w*Mel and *w*Mel*w*AlbB was observed after two weeks (*, p = 0.0159), however, no significant differences could be found at the 4 and 8 week time points. All experiments were conducted using mosquitoes from G8 of the superinfected line and standard error of the mean is indicated.

To determine the mosquito fitness costs of *Wolbachia* superinfection, the longevity (Fig 2) and fecundity and egg survival (Fig 3) of the superinfected line were compared to both uninfected mosquitoes as well as each parental infected line.

#### Longevity

To assess longevity, we compared the survival over time of uninfected, *w*Mel-infected, *w*AlbB-infected and superinfected *Ae*. *aegypti* males (Fig 2A) and females (Fig 2B). Using a Log-rank (Mantel-Cox) as well as a Gehan-Breslow-Wilcoxon test we observed a significant *Wolbachia* strain effect on the survival of both females (df = 3, p ≤ 0.0001) and males (df = 3, p ≤ 0.0001). Superinfected females survived significantly shorter than uninfected mosquitoes (df = 1, p = 0.0034) or mosquitoes infected with either *w*Mel (df = 1, p < 0.0001) or *w*AlbB (df = 1, p < 0.0001). Superinfected females had a mean survival time of 38 days compared to 43.5 days for uninfected females, 47.5 days for *w*Mel-infected females and 50.5 days for *w*AlbB-infected females (Fig 2B). No significant difference in survival between superinfected and *w*Mel-infected males (37 vs 34 days, df = 1, p = 0.16) was observed. However, both *w*Mel-infected and superinfected males survived significantly shorter than both uninfected males (42 days, df = 1, p < 0.0001) and *w*AlbB-infected males (48 days) (df = 1, p < 0.0001) Fig 2A).

#### Fecundity

To assess egg production in the infected and uninfected lines, three independent human blood feeders fed females from each line and single females were subsequently allowed to oviposit on individual pieces of wet filter paper. Total egg laying surface were kept consistent between individual females. From these, 20 egg papers for each blood feeder were randomly selected (60 papers per line) and the egg numbers scored. Using a two-way ANOVA, we first determined that individual blood feeders did not contribute significantly to the observed variation (F = 2.858, p = 0.059) and that the majority of observed variation was derived from the respective lines (F = 9.551, p < 0.0001). All the counts from each line (60 in total) were then combined and statistical differences were determined using a Kruskal-Wallis multiple comparisons test. No significant differences were observed between the superinfected strain and the uninfected or parental strains. In our study design, *w*Mel-infected females laid significantly more eggs than uninfected (p < 0.001) and *w*AlbB-infected (p < 0.001) females (Fig 3A).

#### Egg hatch

The hatch rates of eggs were compared between infected and uninfected lines after 2, 4 and 8 weeks. For each line, approximately 250 females were fed by a single human blood feeder and allowed to oviposit on wet filter papers. The papers were collected, dried and for each storage period, 4 papers per line were hatched. Statistical differences between hatch rates for each line were determined using a two-way ANOVA. We observed a significant effect for both time (F = 20.21, p < 0.0001) as well as mosquito line (F = 76.77, p < 0.0001). Differences between the four lines for each time point are summarized in Fig 3B. In particular, we found that eggs from the superinfected line had significantly lower hatch rates over time than eggs from uninfected females or eggs from *w*AlbB females at all time points. Compared to *w*Mel, we found a small but significant decrease in egg hatch percentage of *w*Mel*w*AlbB infected eggs after 2 weeks. However, no significant differences could be found at the 4 and 8 week time points.

### Comparative susceptibility to DENV infection

**Fig 4:**
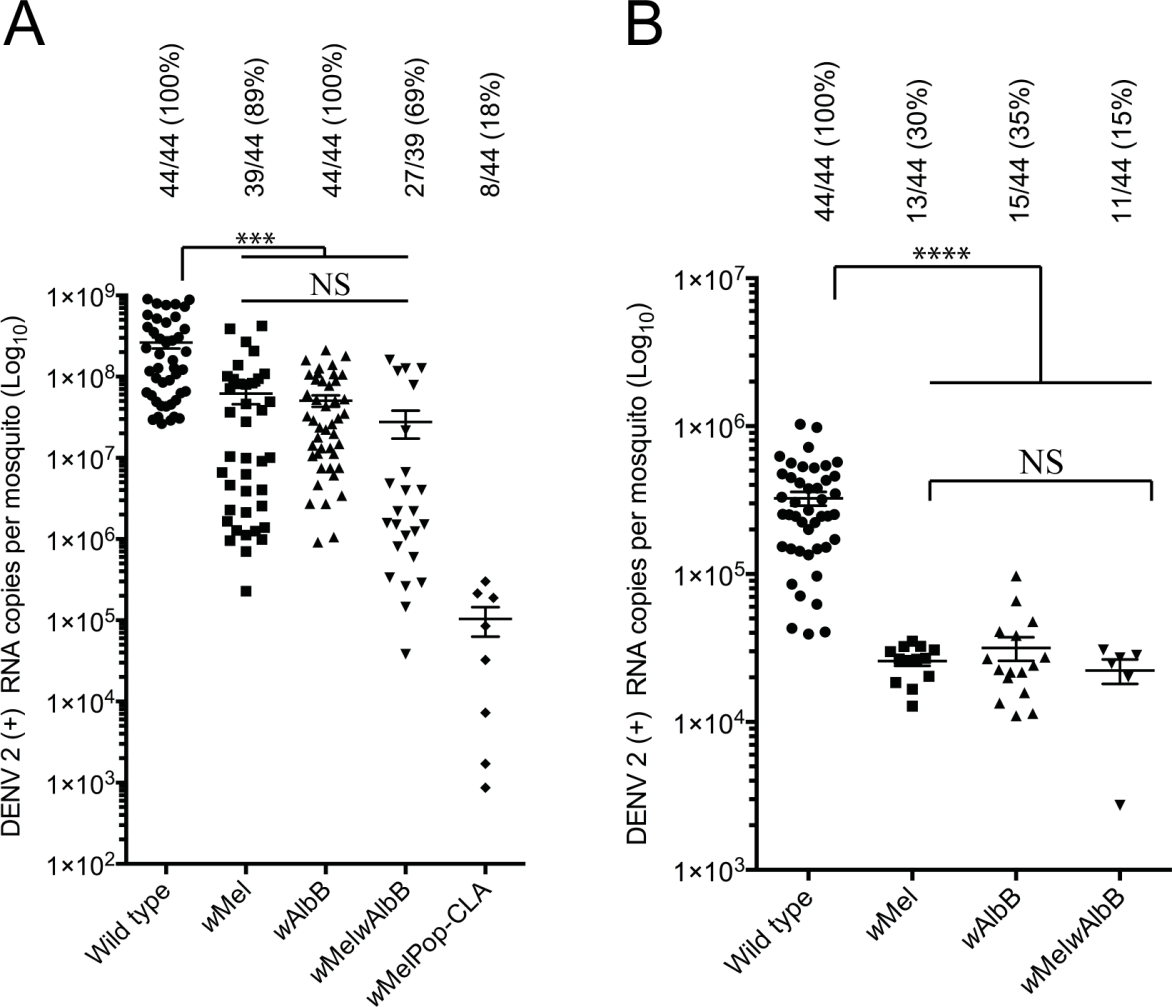
DENV serotype 2 (ET300) genome copies in *Wolbachia*-infected and wild type mosquitoes. (A) DENV-2 was injected at 10^4^ genome copies per ml into the thorax of 3–5 day old female mosquitoes. Virus replication was quantified 7 days post injection in whole mosquito bodies using qPCR. For each mosquito line the mean number of genome copies is plotted and standard error of the mean is indicated. Infection rates are indicated as percentages. (B) DENV-2 was administered in a blood meal at 10^7^ copies per ml. Virus replication was quantified in whole mosquito bodies 14 days post feeding. For each mosquito line the mean number of genome copies is plotted and standard error of the mean is indicated. For both A and B significant differences between wild type and *Wolbachia* infected mosquitoes are indicated by *** (p < 0.0001 and infection rates are indicated as percentages). Zero values were not plotted or included in determining the mean or standard error of the mean.

To test the extent to which DENV replication is relatively inhibited in the *w*Mel*w*AlbB line, we first challenged wild type, *w*Mel, *w*AlbB, *w*MelPop-CLA and *w*Mel*w*AlbB infected mosquitoes with DENV-2 using intrathoracic injections. A DENV-2 strain ET300 was injected at a titre of 10^4^ genome copies/mL and mosquitoes were incubated for 7 days. Positive strand DENV-2 RNA genome copies were detected and quantified in whole mosquito bodies using qRT-PCR. Consistent with previous findings, we saw a significant ~ 1 log reduction of DENV-2 genome copies in *w*Mel and *w*AlbB, whilst in *w*MelPop-CLA mosquitoes, DENV-2 genome copies were dramatically reduced by ~ 4 logs (Fig 4A). No significant differences in DENV-2 copies between the *w*Mel*w*AlbB superinfected line and each of the parental lines were observed (Fig 4A). However, DENV-2 infection rates (calculated as the percentage of DENV-2 infected mosquitoes of the total injected) in *w*Mel*w*AlbB were consistently lower (69%) than both *w*Mel (89%) (Fisher’s exact test, p = 0.034) and *w*AlbB (100%) (Fisher’s exact test, p>0.0001).

We next challenged wild type, *w*Mel, *w*AlbB and *w*Mel*w*AlbB mosquitoes with DENV-2 (ET300) by oral feeding. Defribrinated sheep blood was inoculated with 10^7^ DENV genome copies per ml and 5–6 day old females from each line were allowed to feed for 2 hours using artificial feeders. Fully fed females were selected and incubated for 14 days. Positive strand DENV-2 RNA genome copies were detected and quantified in whole mosquito bodies using qPCR. We found a significant ~1.5 log reduction in DENV-2 genome copies in *w*Mel, *w*AlbB as well as *w*Mel*w*AlbB mosquitoes compared to wild type. No significant difference in DENV-2 genome copies between the three *Wolbachia*-infected lines were found (Fig 4B). We did observe non-significant, lower DENV infection rates in the *w*Mel*w*AlbB infected line (15%) as compared to the *w*Mel (30%) (Fisher’s exact test, p = 0.41) and *w*AlbB (35%) (Fisher’s exact test, p = 0.24) infected lines (Fig 4B).

We then assessed the susceptibility of wild type, *w*Mel and *w*Mel*w*AlbB mosquitoes to DENV infection after feeding on human viremic blood from 43 dengue patients admitted to the Hospital for Tropical Diseases in Ho Chi Minh City, Vietnam. Two feeds were excluded from analysis; a flow chart describing the number of blood fed mosquitoes, their survival and the final cohorts for analyses are described in S2 Fig. The characteristics of the 41 blood donor patients are shown in S1 Table. DENV-1 and DENV-4 were the predominant infecting serotypes in the patient donors (88% of infectious feeds).

**Fig 5:**
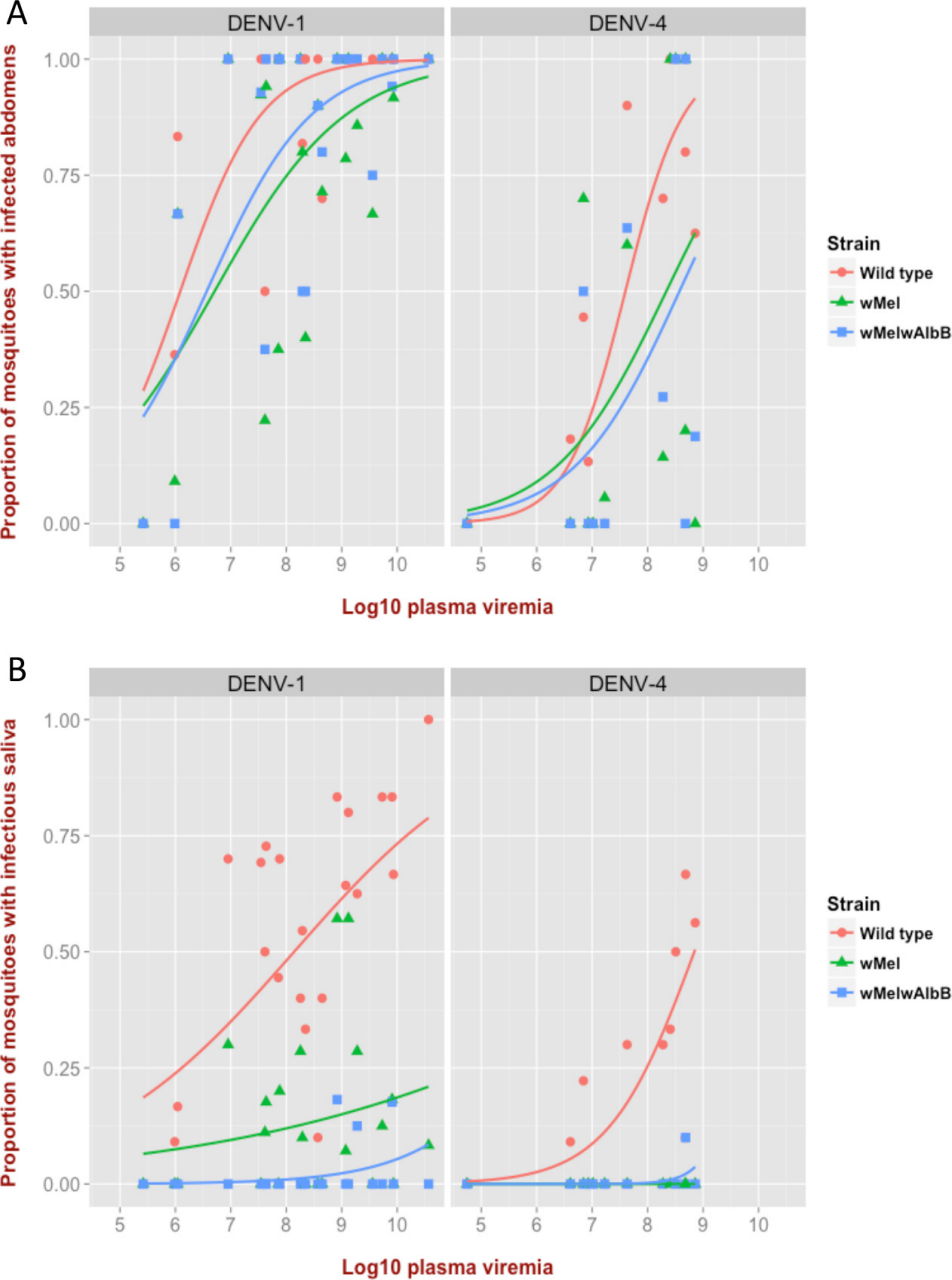
Proportion of mosquitoes infected with DENV after blood-feeding on 36 dengue patient blood samples, as a function of plasma viremia, and stratified by serotype. Lines represent fits from logistic regression to the data. All three strains were assessed for every feed; for DENV-1 there were 23 blood feeds, and 13 for DENV-4. DENV-2 and DENV-3 are not represented due to small sample sizes (n = 3 and n = 2 respectively). (A) Each point represents the proportion of mosquitoes with a DENV-infected abdomen, stratified by strain, and pooled across all time points. (B) The proportion of mosquitoes, pooled across all time points, that expectorated infectious DENV in their saliva.

The *w*Mel and superinfected *w*Mel*w*AlbB lines had lower frequencies of DENV infection than wild-type mosquitoes in abdomens and saliva (Fig 5 and Table 3). Across all time points, a total of 42.65% of wild-type mosquitoes had infectious saliva versus 6.57% for *w*Mel and 2.89% for *w*Mel*w*AlbB (adjusted odds ratio (OR) 0.065; 95% CI = 0.038–0.112; p <0.001 for *w*Mel, and OR 0.025; 95% CI = 0.014–0.043; p < 0.001 for *w*Mel*w*AlbB versus wild-type) (Table 3). *w*Mel*w*AlbB further reduced the risk of females having infectious saliva compared to *w*Mel-infected females (OR = 0.377; 95% CI = 0.196–0.725; p = 0.003).

**Table 3:** Marginal logistic regression models for the risk of viral infection in the abdomen tissue, and for infectious saliva.

	ABDOMEN					SALIVA				
	OR	Lower CI	Upper CI	p-value		OR	Lower CI	Upper CI	p-value	
**Patients’ viremia (+1 log 10 copies/ml)**	3.070	1.903	4.952	0.000	***	1.920	1.485	2.481	0.000	***
**DENV-1 (reference)**										
**DENV-2**	1.090	0.278	4.278	0.901		0.406	0.212	0.778	0.007	**
**DENV-3**	0.108	0.021	0.561	0.008	**	0.158	0.033	0.753	0.021	*
**DENV-4**	0.106	0.027	0.422	0.001	**	0.393	0.202	0.765	0.006	**
**Day 10 (reference)**										
**Day 14**	0.978	0.809	1.181	0.814		2.508	1.713	3.673	<0.001	***
**Day 18**	0.861	0.644	1.152	0.315		2.356	1.417	3.918	0.001	**
**WT (reference)**										
***w*Mel** ^**#**^	0.270	0.163	0.449	<0.001	***	0.065	0.038	0.112	<0.001	***
***w*Mel*w*AlbB** ^**#**^	0.337	0.211	0.539	<0.001	***	0.025	0.014	0.043	<0.001	***

Results indicate that both *Wolbachia* strains significantly reduce the likelihood of mosquitoes becoming infected.

OR = Odds ratio, CI = Confidence intervals, WT = Wild type

* = *p* < 0.05

** = *p* < 0.01

*** = *p* < 0.001.

^#^ Comparison between *w*Mel and *w*Mel*w*AlbB give OR = 1.247 (95% CI = 0.927 to 1.678, p = 0.145) for abdomen and OR = 0.377 (95% CI = 0.196 to 0.725, p = 0.003) for saliva, indicating that the risk of viral infection in saliva (but not in abdomens) is significantly lower for *w*Mel*w*AlbB infections compared to *w*Mel.

**Fig 6:**
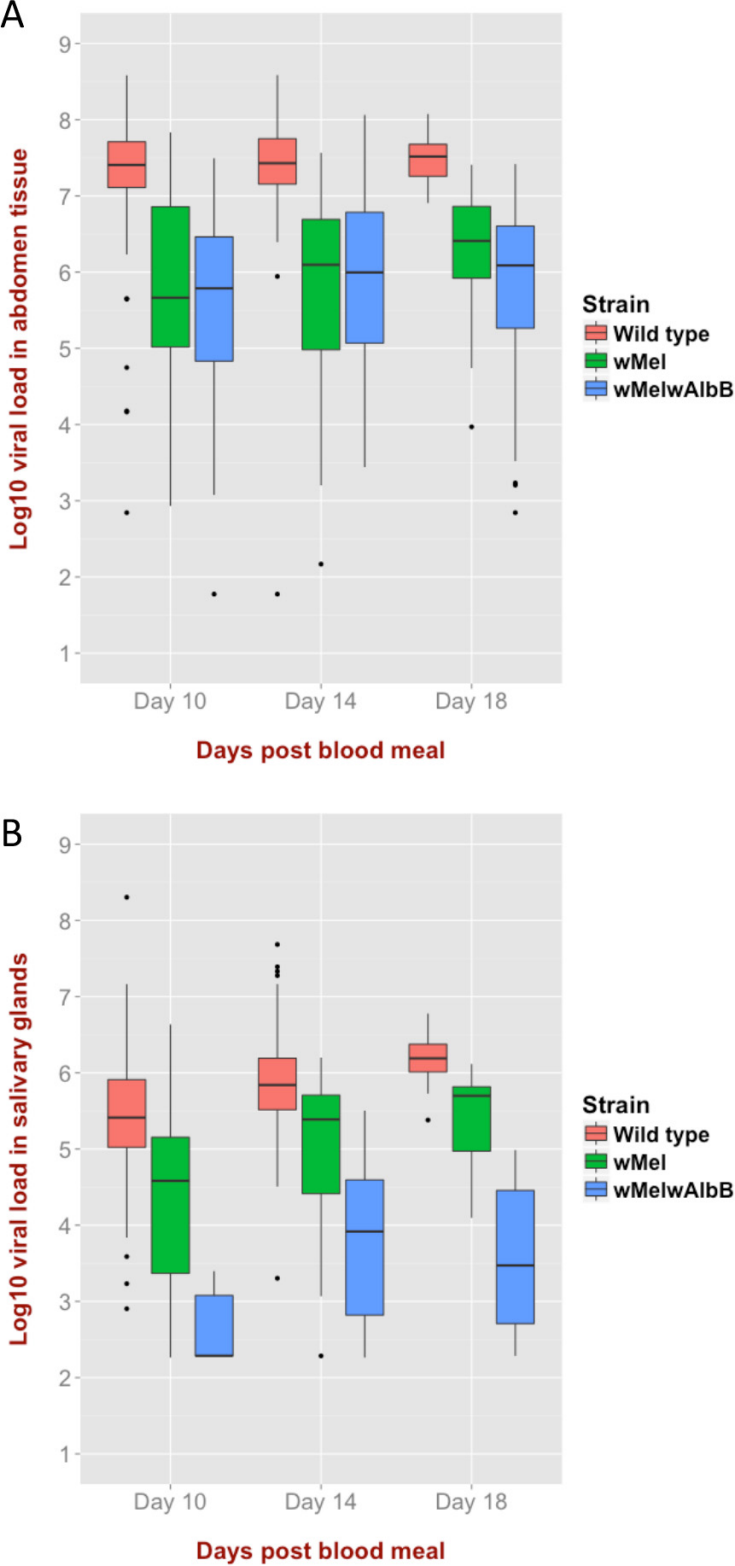
Box plots representing the viral load of DENV in tissues of DENV-infected mosquitoes. Virus was detected at days 10, 14 and 18 post exposure to 41 patient-derived infectious blood meals. (A) Abdomen tissue. (B) Salivary gland tissue. As detailed in S2 Table, *w*Mel and *w*Mel*w*AlbB are associated with significantly lower viral loads in abdomen tissue and salivary glands compared to wild type, and *w*Mel*w*AlbB is associated with significantly lower viral loads in salivary glands compared to *w*Mel.

In addition, *Wolbachia*-infected mosquito strains also had significantly lower concentrations of DENV RNA in their abdomen and salivary gland tissues compared to wild-type mosquitoes (Fig 6A and 6B and S2 Table). *w*Mel*w*AlbB blocked DENV infection in the salivary glands more efficiently than *w*Mel (Fig 6B). Collectively, these data generated using clinically-relevant virus challenge methods, suggest that the *w*Mel*w*AlbB strain delivers an incrementally improved DENV blocking phenotype compared to *w*Mel.

## Discussion

*Wolbachia* has been shown to inhibit pathogen replication in both natural and transinfected insects [9–12, 18, 20]. Combined with *Wolbachia’s* remarkable evolutionary adaptations to ensure rapid spread and transmission, [5, 28] this bacterium holds promise as an effective biocontrol agent against mosquito-borne diseases such as dengue [20]. Trials with the *w*Mel strain of *Wolbachia* have shown its establishment and spread in both semi-field [12] and wild populations of *Aedes aegypti* mosquitoes [19]. However, not all *Wolbachia* strains are suitable for use in biocontrol strategies. The virulent *w*MelPop-CLA strain, for example, results in greater overall inhibition of DENV replication in adult female mosquitoes than the avirulent *Wolbachia* strains, but imparts significantly higher fitness costs [11, 12, 29]. Preliminary trials in Australia and Vietnam in which the *w*MelPop-CLA strain was released into wild mosquito populations indicate that these fitness costs prevented successful establishment [30].

Modelling projections suggest the establishment of *Wolbachia* strains in dengue endemic settings will result in a substantial reduction in disease burden [31]. The persistence of an inhibitory effect on DENV replication within wild *Wolbachia*-infected mosquitoes will be key to the success of any release program. Laboratory vector competence experiments with field (F1) *w*Mel-infected *Ae*. *aegypti* mosquitoes, collected one year following field release, indicated very low levels of DENV replication and dissemination [32], demonstrating the persistence of the virus inhibition phenotype. The potential evolution of DENV resistance to *Wolbachia’s* inhibitory effects must be considered if this biocontrol strategy can be sustainable on a long-term basis. However, the ability to predict the likelihood of resistance development in virus populations will require a greater understanding of the mechanisms of *Wolbachia*-mediated viral inhibition. Host immune stimulation has been shown to result in antiviral effects in *Ae*. *aegypti* [10, 25, 33] but this is not universal for all *Wolbachia*-mediated antiviral inhibition [34–36]. The density and tissue tropism of *Wolbachia* strains in insect hosts appears to be the most important factors [12, 37, 38] and competition for shared host resources such as cholesterol has been shown to influence the strength of *Wolbachia*-induced antiviral effects [17]. High density *Wolbachia* strains in Drosophila flies provide strong inhibitory effects on insect viruses despite a long-term evolutionary association [11, 39]. Thus, the non-specific nature of the anti-viral environment in *Wolbachia-*infected *Ae*. *aegypti* tissues, coupled with the dominant evolutionary process of purifying selection in DENV populations[40], such that minor variant viruses that arise within individual hosts are lost because they are not infectious to both humans and mosquitoes, creates significant barriers to the emergence of DENV strains that are resistant to *Wolbachia*. Nonetheless, the association between density and viral inhibition in these natural *Wolbachia*-host endosymbiotic relationships suggest resistance is less likely to develop for *Wolbachia* strains that grow to high densities in transinfected insect hosts. Therefore, a superinfection that results in a cumulative higher density *Wolbachia* infection would be predicted to reduce the potential for DENV resistance development in *Ae*. *aegypti*.

In the event DENV does evolve resistance to either the *w*Mel or *w*AlbB strains in wild mosquito populations, one potential option would be to release a superinfected line that would ‘sweep over’ the existing single infection. For this resistance management strategy to be effective, favourable CI spread dynamics would be needed for a superinfected line to replace existing single *Wolbachia* infections in wild mosquito populations. The crossing patterns induced by *w*Mel*w*AlbB (Table 2) indicate that either the *w*Mel or *w*AlbB strain could be replaced by a superinfection in wild mosquito populations.

The density of *Wolbachia* strains in transinfected *Ae*. *aegypti* mosquitoes is also correlated with mosquito fitness costs [12]. The additive density of *Wolbachia* strains in the superinfected line, measured at G18 when the line was stable, was comparable to the virulent *w*MelPop-CLA strain (Fig 1B). Despite the superinfected line resulting in a cumulative high density *Wolbachia* infection, the effects on the majority of mosquito fitness parameters were very similar to that observed for the single infected *w*Mel line. Under laboratory conditions superinfected males and females had a marginally shorter adult lifespan than uninfected wild type mosquitoes (~10% reduction). The observed effects on adult mosquito longevity of the superinfected line are significantly less than those for the virulent *w*MelPop-CLA strain, which reduces the lifespan of adult *Ae*. *aegypti* mosquitoes by approximately ~50% [9].

In our study, no differences in the number of eggs laid by females (fecundity) from the superinfected line compared to *w*Mel, *w*AlbB or wild type mosquitoes were observed. Under semi-field conditions, the virulent *w*MelPop-CLA strain reduced fecundity of *Ae*. *aegypti* females by ~60% [12], which may have contributed to the inability of this strain to invade wild mosquito populations [41]. Minimal fecundity costs should increase the potential of the superinfected line to ‘sweep over’ existing single infections in wild mosquito populations. In contrast, survival of eggs from superinfected females during periods of embryonic quiescence was significantly lower than either parental line or wild type mosquitoes. Following two months of storage, ~50% of superinfected eggs were still viable. Although the hatch rates for the superinfected line were lower than that observed for the *w*AlbB- infected line, the hatch rates were very similar to that of the *w*Mel infected line. Furthermore, hatch rates are still within the average 2-month survival rates (40–60%) for *Ae*. *aegypti* eggs during dry seasons [42, 43]. Further experiments under semi-field conditions will be needed to fully determine if the effect on embryonic quiescence is likely to impact the ability of the superinfected line to invade uninfected wild mosquito populations. The results of field releases to date (using *w*Mel) suggest this is unlikely to be a major obstacle to establishing superinfections in the field. The *w*Mel strain successfully invaded wild mosquito populations [19] and the infection remains stable in these release areas [44] despite the observed reduction on embryo hatch rates under laboratory conditions.

The release of a superinfected line for virus resistance management would require the co-infection to provide strong inhibitory effects on DENV replication. Vector competence experiments carried out under laboratory conditions indicated all *Wolbachia* lines significantly reduced DENV replication as previously reported [12, 25], however the superinfected line provided the greatest resistance. After oral feeding on fresh human viremic blood, the most relevant model to assess mosquito susceptibility to DENV, very few superinfected mosquitoes had infectious virus in their saliva and viral RNA concentrations were substantially reduced in mosquito tissues. These data give reassurance that any population replacement strategy with the superinfected line would be expected to deliver stronger inhibition of DENV transmission than is conferred by *w*Mel.

In summary, the generation and characterisation of a superinfected line with the desired phenotypic effects to replace single wild infections provides a potential mechanism to overcome the emergence of DENV resistance. Both *Wolbachia* strains are stably maintained in the line with minimal mosquito fitness effects. Importantly, DENV replication is inhibited to a greater extent in the superinfected line compared to both parental lines. The observed CI phenotype induced by the superinfected line is of particular significance as it would enable the line to be released “on top of” existing *w*Mel or *w*AlbB field releases in dengue endemic areas.

## Materials and Methods

### Mosquito colonies and lines

*Wolbachia*-uninfected *Ae*. *aegypti* eggs were collected from Cairns (Queensland, Australia) in 2013 (JCU wild type). The *Wolbachia*-infected *w*Mel and *w*AlbB mosquito lines have been described previously [12, 25]. All *Ae*. *aegypti* mosquitoes were reared and maintained as described in [9] with the following modification. For hatching, eggs were placed in hatching water (distilled H_2_O, boiled and supplemented with 50 mg/L fish food [Tetramin]) and allowed to hatch for 24 h. Larvae were subsequently reared at a set density of ~150 in 3 L of distilled water as described in [9]. To prevent genetic drift between wild type and the *Wolbachia* infected mosquito lines used for analyses, females from each generation of the infected lines were backcrossed with a small proportion (10%) of uninfected field collected male mosquitoes.

Embryonic microinjection, isofemale line establishment and selection for stably-infected lines were done as previously described [9]. In short, the *w*Mel strain was purified from *w*Mel-infected mosquitoes and microinjected into the posterior-pole of *w*AlbB-infected preblastoderm embryos using methodology previously described [12]. Surviving G0 adult females (~600) from microinjection were mated to wild type males and blood fed for oviposition of the G1 generation. G0 females that laid fertile egg batches were screened using quantitative PCR as described by [17] and primers specific for *w*Mel (forward primer: 5’-CAAATTGCTCTTGTCCTGTGG-3’, reverse primer: 5’-GGGTGTTAAGCAGAGTTACGG-3’) and *w*AlbB (forward primer: 5’-CCTTACCTCCTGCACAACAA-3’, reverse primer: 5’-GGATTGTCCAGTGGCCTTA-3’). For each sample, quantitative PCR amplification of DNA was performed in duplicate with a LightCycler 480 II Instrument (Roche) using LightCycler 480 SYBR Green I Master (Roche) according to the manufacturer’s protocol. From the ~600 females screened, 21 *w*Mel positives were identified and pooled into two lines. The female progeny from both lines of superinfected females were mated to uninfected field-males for 5 generations (G_0_-G_4_) before the lines were considered stably infected with both strains of *Wolbachia*. One line was selected for further characterisation.

### *Wolbachia* distribution & density

*Wolbachia* density and distribution in the superinfected line was compared to each of the parental strains using qPCR and fluorescence *in situ* hybridisation (FISH). Quantitative PCR to determine the total relative *Wolbachia* densities of infected lines was performed as described by [17] using primers specific to the gene coding for the *Wolbachia* surface protein (*wsp*) (forward primer 5’ GCATTTGGTTAYAAAATGGACGA-3’, reverse primer 5’- GGAGTGATAGGCATATCTTCAAT-3’), as well as the *Ae*. *aegypti rps17* gene (forward primer 5’-TCCGTGGTATCTCCATCAAGCT-3’, reverse primer: 5’-CACTTCCGGCACGTAGTTGTC-3’).

*Wolbachia* was localized in sections of paraffin-embedded 5–7 day old female mosquitoes by FISH, as described in [10], except that only one probe against 16S rRNA was used against each strain and their concentration was increased by 10-fold to improve the signal. *w*Mel was detected using the probe MelPopW6: 5’-GCTTAGCCTCGCGACTTTGCAG-3’, labelled with Alexa 594 dye (red), whereas *w*AlbB was localized using AlbBW5: 5’-CTTAGGCTTGCGCACCTTGCAA-3’, labelled with Alexa 488 dye (green). 16S rRNA is highly conserved between *w*Mel and *w*AlbB, therefore the probe was designed against a part of the gene that includes several SNPs. We confirmed the specificity and lack of cross-reactivity of each probe by testing them against the single infected lines (*w*Mel and *w*AlbB). Both probes were added simultaneously to the *w*Mel, *w*AlbB and *w*Mel*w*AlbB mosquito sections in order to obtain the images. DAPI was also used to stain total DNA.

### Fitness determinants

#### Longevity

The adult lifespan of *Ae*. *aegypti* superinfected with both *w*Mel and *w*AlbB was compared to wild type, *w*Mel, and *w*AlbB-infected lines. For each mosquito line used, 6x 500 mL mesh covered plastic containers with 20 virgin males and 6x containers with 20 virgin females were incubated as described in [9]. Mosquitoes were fed on a 10% sucrose solution and live mosquitoes were counted daily until all the mosquitoes were dead. Survival curves were compared using a Log-rank (Mantel-Cox) as well as a Gehan-Breslow-Wilcoxon test.

#### Fecundity

Five day old *Ae*. *aegypti* females from each mosquito line used (wild type, *w*Mel, *w*AlbB and *w*Mel*w*AlbB) were fed on the arm of one human volunteer. This was repeated two more times with different human volunteers for each repeat (each mosquito line in a single repeat were fed by the same volunteer) (Monash University human ethics permit no. CF11/0766-2011000387). Females were aspirated into individual tubes one-day post blood feeding and allowed to oviposit on wet filter paper. For each line, 20 egg laying females per blood feeder (60 in total) were randomly selected. The eggs were matured for three days and then counted. The counts for each line were combined and compared using a Kruskal-Willis rank-sum test.

#### Fertility

Approximately 250 females from each line were fed by the same human blood feeder and allowed to oviposit on wet filter paper. The egg papers were dried slowly under controlled humidity (80%) and temperature (26°C) for 5–7 days and counted as described. From each line, four egg papers were hatched in individual plastic trays as previously described at 2, 4 and 8 week intervals. Papers were removed after 24 h and placed in trays with fresh hatching water to allow any remaining viable eggs to hatch. For each egg paper, hatched second instar larvae were counted to determine egg hatch rate.

#### CI and maternal transmission

To investigate if there was any CI caused between the superinfected *w*Mel*w*AlbB mosquitoes with either the uninfected (JCU wild type) or singly infected (*w*Mel and *w*AlbB) mosquitoes, we conducted reciprocal crosses. Ten crosses were set up in cages with 50 virgin females and 50 males each between *w*Mel*w*AlbB x JCU, *w*Mel x JCU, *w*AlbB x JCU, *w*Mel*w*AlbB x *w*Mel, *w*Mel*w*AlbB x *w*AlbB. In addition, 4 self crosses of *w*Mel*w*AlbB, *w*Mel, *w*AlbB and JCU with 50 males and females each were also set up as controls.

Groups were allowed to mate for 3–5 days before the females were blood fed. All females were blood fed on the arms of one human volunteer. Two days after blood feeding single females were set up for oviposition. 24–48 h post oviposition, eggs were dried slowly under controlled humidity (80%) and temperature (26°C) for 5–7 days. Eggs were counted and hatched as described, all the hatched larvae were counted within 24–48 h of hatch and the mean hatch percentage was calculated.

To determine maternal transmission rates 100 virgin females of G12, G14 and G17 of the *w*Mel*w*AlbB line were outcrossed with 100 uninfected JCU wild type males in cages and allowed to oviposit. The eggs were hatched as described and the progeny (88 for G12, 64 for G13 and 64 for G17) was screened for *w*Mel and *w*AlbB.

### Susceptibility to DENV-2 infection

The propagation and maintenance of dengue virus serotype 2 (DENV-2) ET300 was carried out as previously described [18]. For adult microinjections, 40 *Ae*. *aegypti* female mosquitoes were anesthetized by briefly exposing them to -20°C. The mosquitoes were subsequently injected intrathoracically with 50 nL of virus solution (10^4^ genomic copies/ml in RPMI [Sigma-Aldrich] media) using a pulled glass capillary and a handheld microinjector (Nanoject II, Drummond Sci.). Injected mosquitoes were incubated for 7 days (40 mosquitoes per cup) at 26°C with 65% relative humidity and a 12h light/dark cycle. For feeding experiments with DENV-2 (ET300) infected blood, 80 *Ae*. *aegypti* female mosquitoes were placed in 500 mL plastic containers, starved for 25 hours and allowed to feed on a 50:50 mixture of defibrinated sheep blood and tissue culture supernatant containing 10^7^ genome copies/mL of DENV-2. Feeding was done through a piece of desalted porcine intestine stretched over a water-jacketed membrane feeding apparatus preheated to 37°C for approximately three hours. Fully engorged mosquitoes were placed in 500 mL containers and incubated for 14 days at 26°C with 65% relative humidity and a 12h light/dark cycle.

To quantify DENV-2 genomic copies, total RNA was isolated from DENV-2 injected mosquitoes using the Nucleospin 96 RNA kit (Macherey-Nagel). DENV-2 qPCR analysis was done using cDNA prepared from individual mosquitoes according to [10]. Statistical significance for differences in DENV titres between treatments was determined using a one-way ANOVA with Tukey’s multiple comparison tests (Graph Pad Prism 6c).

### Oral challenge with human viremic blood

Cohorts of 3–5 day old mosquitoes were allowed to feed on fresh, viremic blood from 43 NS1 rapid test-positive patients admitted to the Hospital for Tropical Diseases, in Ho Chi Minh City, Vietnam. Mosquitoes were fed via membrane feeders for a maximum of 1 hour. Fully engorged mosquitoes were placed in 250 mL containers and incubated at 28°C/80% humidity with a 12h light/dark cycle. Mosquitoes were harvested from each blood fed cohort 10, 14 and 18 days later. Detection of infectious virus in the saliva of each mosquito was as described previously [45]. Statistical analyses were performed with the statistical software R, version 3.1.3 (R Foundation for Statistical Computing, Vienna, Austria). Marginal regression models for binary (infected/uninfected mosquitoes) and continuous (tissue viral load) outcomes were fitted using generalized estimating equations with working exchangeable correlation structure to account for potential within-patient correlation.

### Ethics statement

Blood feeding by volunteers (Monash University human ethics permit no CF11/0766-2011000387) for this study was approved by the Monash University Human Research Ethics Committee (MUHREC). All adult volunteers provided informed written consent; no child participants were involved in the study.

The protocol for feeding mosquitoes with viremic human blood was reviewed and approved by the Ethics Committee of Hospital for Tropical Diseases (HTD), Ho Chi Minh City, Vietnam (approval number CS/ND/12/16), and the Oxford University Tropical Research Ethics Committee (OxTREC) (approval number OxTREC 30–12). All enrolled subjects provided informed written consent.

## Supporting Information

S1 Fig Localisation of *w*Mel (red) and *w*AlbB (green) in the Midgut epithelia, Thoracic ganglia, Salivary gland and Malpighian tubules of the superinfected *Ae*. *aegypti* line. (TIF)

S2 Fig Flowchart showing numbers of *Aedes aegypti* analysed for susceptibility to DENV infection after exposure to patient-derived blood meals. (TIF)

S1 Table Baseline patient characteristics for the 41 successful infectious feeds performed using viremic human blood. (DOCX)

S2 Table Marginal multiple linear regression models for viral load (log10 copies/ml) in abdomens and salivary glands depending on covariates. Only infected abdomen or salivary glands were included. Results indicate that both *Wolbachia* strains significantly reduce the concentration of DENV in respective infected tissues. Coef = Regression coefficients, CI = Confidence intervals, WT = Wild type, * = *p* < 0.05, ** = *p* < 0.01, *** = *p* < 0.001. ^#^ Comparison between *w*Mel and *w*Mel*w*AlbB give coefficients of -0.147 (95% CI -0.549 to 0.255, p = 0.474) for abdomen and -1.546 (95% CI = -1.848 to -1.245, p < 0.001) for salivary glands, indicating lower viral loads in salivary glands of *w*Mel*w*AlbB infected females compared to the *w*Mel infection.
(DOCX)
