# Supplementary figures and images for: Establishment of a Wolbachia Superinfection in Aedes aegypti Mosquitoes as a Potential Approach for Future Resistance Management
Source: PLoS Pathog. 2016 Feb 18;12(2):e1005434. doi: 10.1371/journal.ppat.1005434 (PMC4758728; doi:10.1371/journal.ppat.1005434)

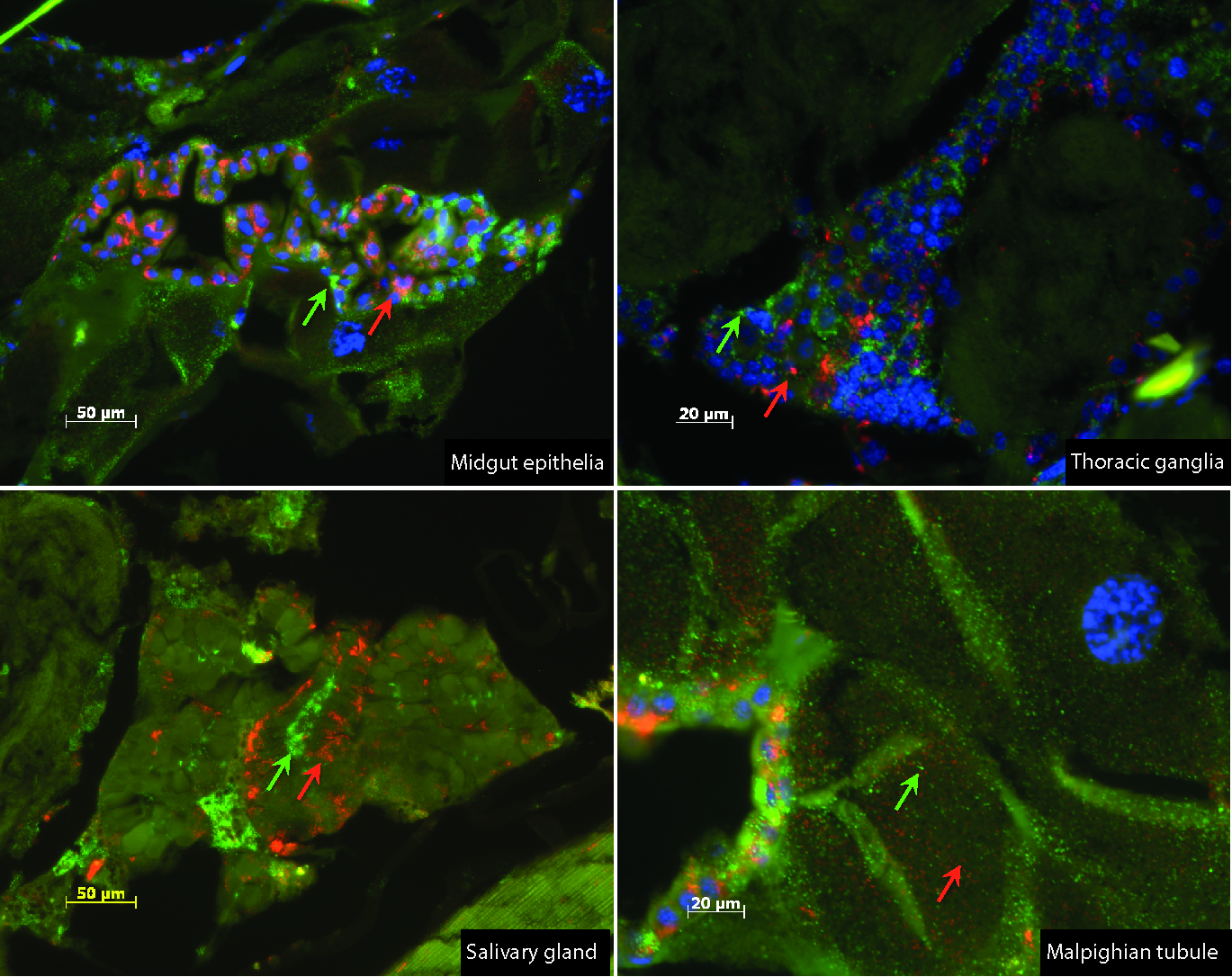

Supplement: S1 Fig — (TIF) [file ppat.1005434.s001.tif]

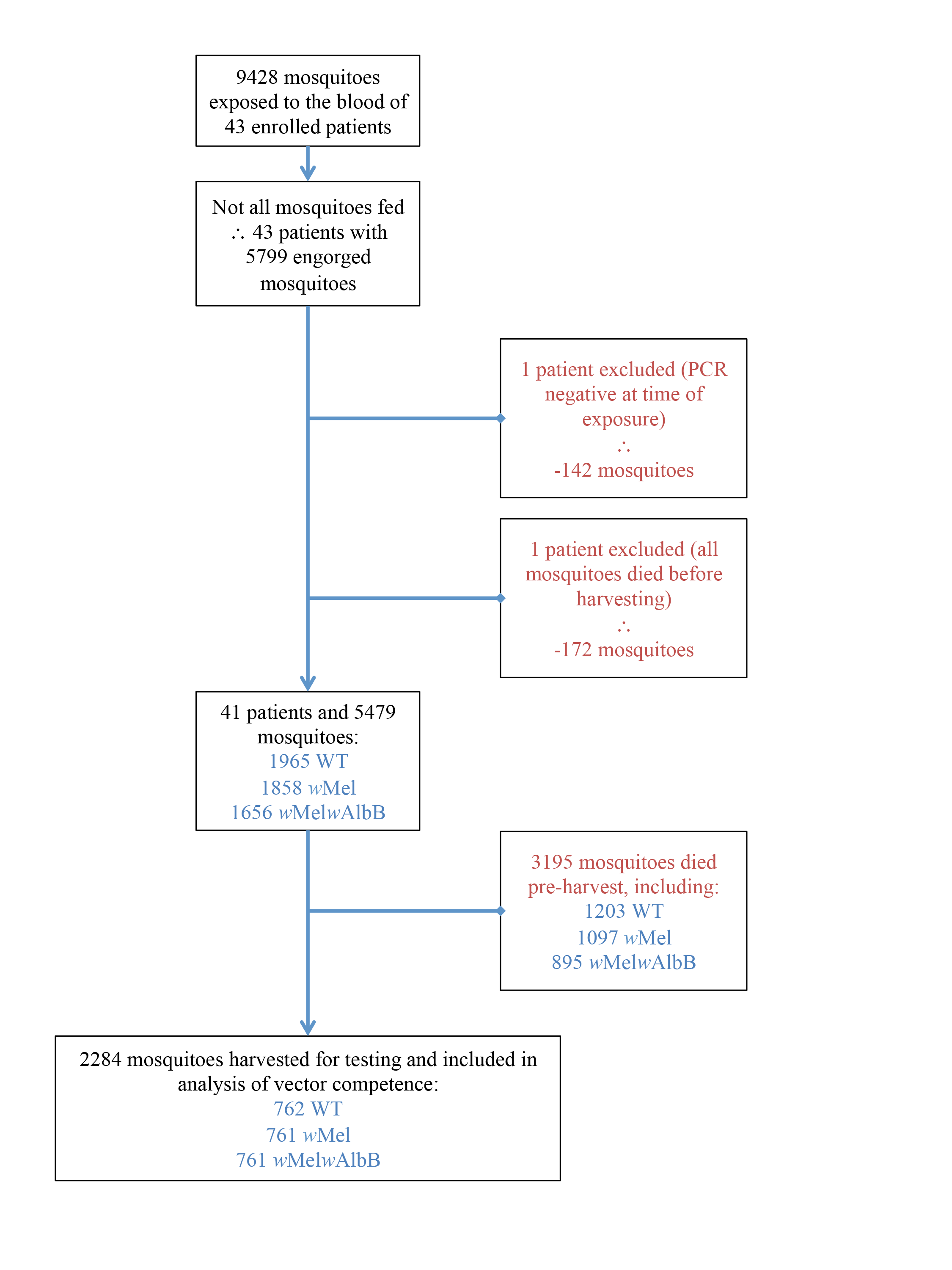

Supplement: S2 Fig — (TIF) [file ppat.1005434.s002.tif]
