# Supplementary material for: Establishment of a Wolbachia Superinfection in Aedes aegypti Mosquitoes as a Potential Approach for Future Resistance Management
Source: PLoS Pathog. 2016 Feb 18;12(2):e1005434. doi: 10.1371/journal.ppat.1005434 (PMC4758728; doi:10.1371/journal.ppat.1005434)
Supplement: S1 Table — (DOCX) [file ppat.1005434.s003.docx]

|  |  |  | **DENV-1 (n=23)** | **DENV-2 (n=3)** | **DENV-3 (n=2)** | **DENV-4 (n=13)** | **Total (n=41)** |
| --- | --- | --- | --- | --- | --- | --- | --- |
| **Age** | years | median (IQR) | 26 (18-52) | 22 (20-23) | 29 (25-33) | 28 (18-40) | 27 (18-52) |
| **Sex** | female | n (%) | 10 (43%) | 1 (33.3%) | 2 (100%) | 5 (38%) | 18 (44%) |
|  | male | n (%) | 13 (57%) | 2 (66.7%) | 0 (0%) | 8 (62%) | 23 (56%) |
| **Log 10 viremia** | | median (range) | 8.35 (5.42-10.57) | 6.48 (6.26-7.13) | 7.22 (6.4-8.04) | 7.63 (4.74-8.86) | 8.04 (4.74-10.57) |
