# Supplementary material for: Establishment of a Wolbachia Superinfection in Aedes aegypti Mosquitoes as a Potential Approach for Future Resistance Management
Source: PLoS Pathog. 2016 Feb 18;12(2):e1005434. doi: 10.1371/journal.ppat.1005434 (PMC4758728; doi:10.1371/journal.ppat.1005434)
Supplement: S2 Table — Only infected abdomen or salivary glands were included. Results indicate that both Wolbachia strains significantly reduce the concentration of DENV in respective infected tissues. Coef = Regression coefficients, CI = Confidence intervals, WT = Wild type, * = p < 0.05, ** = p < 0.01, *** = p < 0.001. # Comparison between wMel and wMelwAlbB give coefficients of -0.147 (95% CI -0.549 to 0.255, p = 0.474) for abdomen and -1.546 (95% CI = -1.848 to -1.245, p < 0.001) for salivary glands, indicating lower viral loads in salivary glands of wMelwAlbB infected females compared to the wMel infection. (DOCX) [file ppat.1005434.s004.docx]

|  | **ABDOMEN** | | | | |  | **SALIVARY GLANDS** | | | | |
| --- | --- | --- | --- | --- | --- | --- | --- | --- | --- | --- | --- |
|  | **Coef** | **Lower CI** | **Upper CI** | **p-value** | |  | **Coef** | **Lower CI** | **Upper CI** | **p-value** | |
| **Patients’ viremia**  **(+1 log 10 copies/ml)** | 0.031 | -0.155 | 0.217 | 0.746 |  |  | 0.113 | -0.073 | 0.299 | 0.233 |  |
| **DENV-1 (reference)** |  |  |  |  |  |  |  |  |  |  |  |
| **DENV-2** | -0.896 | -2.108 | 0.315 | 0.147 |  |  | 0.065 | -1.120 | 1.249 | 0.915 |  |
| **DENV-3** | -0.239 | -0.499 | 0.021 | 0.072 |  |  | 0.440 | 0.160 | 0.721 | 0.002 | ** |
| **DENV-4** | 0.217 | -0.280 | 0.713 | 0.393 |  |  | 0.858 | 0.449 | 1.268 | <0.001 | *** |
| **Day 10 (reference)** |  |  |  |  |  |  |  |  |  |  |  |
| **Day 14** | 0.140 | -0.026 | 0.305 | 0.098 |  |  | 0.662 | 0.470 | 0.854 | <0.001 | *** |
| **Day 18** | -0.004 | -0.304 | 0.297 | 0.981 |  |  | 0.913 | 0.697 | 1.130 | <0.001 | *** |
| **WT (reference)** |  |  |  |  |  |  |  |  |  |  |  |
| ***w*Mel ^#^** | -1.541 | -1.914 | -1.167 | <0.001 | *** |  | -1.013 | -1.376 | -0.649 | <0.001 | *** |
| ***w*Mel*w*AlbB ^#^** | -1.688 | -1.997 | -1.379 | <0.001 | *** |  | -2.559 | -2.906 | -2.211 | <0.001 | *** |
